# A Fast Testing Method to Objectively Quantify the Stiffness of Stability Boots

**DOI:** 10.1155/2015/595708

**Published:** 2015-12-10

**Authors:** Simon Bürgi, Judith Roost, Marco R. Hitz, Peter Schwilch, William R. Taylor, Silvio Lorenzetti

**Affiliations:** Institute for Biomechanics, ETH Zurich, Leopold-Ruzicka-Weg 4, 8093 Zürich, Switzerland

## Abstract

Stability boots can protect the ankle ligaments from overloading after serious injury and facilitate protected movement in order to aid healing of the surrounding soft tissue structures. For comparing different stability shoe designs and prototypes, a reliable and fast testing method (FTM) is required. The aim of this study was to assess the reliability of a novel custom-built device. Six different stability boots were tested in a novel device that allowed body weight to be taken into account using a pneumatic actuator. The fixation of the boots was controlled using two air pad pressure sensors. The range of motion (RoM) was then assessed during 5 trials at physiological ankle joint torques during flexion/extension and inversion/eversion. Furthermore the intraclass correlation coefficient ICC was determined to assess the repetitive reliability of the testing approach. The measured ankle angles ranged from 3.4° to 25° and proved to be highly reliable (ICC = 0.99), with standard deviations <9.8%. Comparing single trials to one another resulted in a change of 0.01° joint angle, with a mean error of 0.02°. The FTM demonstrates that it is possible to reliably measure the ankle joint RoM in both the sagittal and frontal planes at controlled torque levels, together with the application of body weight force.

## 1. Introduction

For active children and adults, the sprained ankle is the most common musculoskeletal injury treated by physicians [1]. Recovering from a sprained ankle can take considerable time, and, in certain cases, lead to long term functional deficits [2]. Protecting the ankle ligaments from overloading after serious injury is necessary in order to achieve complete healing of the structures. Beside bracing, fixing, and taping, stability boots are now commonly used in the rehabilitation of patients who have suffered serious ankle ligament injuries [3–5]. The main characteristic of a stability boot is its ability to reduce the range of motion (RoM) of the ankle joint while maintaining the patient's mobility [3, 6].

Currently, few methods exist to objectively compare different stability boots regarding their stiffness and effect on the RoM at the ankle joint. Previous studies have compared different orthotics or shoes using mechanical devices but also by examining rehabilitation progress and functional outcome of the joint. van Jaarsveld and coworkers investigated stiffness properties of prosthetic feet using a robot [7]. By pressing a plate onto the prosthetic foot in different positions, rotational motion of the ankle joint was simulated throughout the whole stance phase. In a similar manner, Cikajlo and Matjačić build a device to assess boot stiffness by pressing the boot onto a slope and adjusting the slope angles and location of first contact to simulate ankle kinematics during stance phase [8]. Bregman and coworkers manually applied ankle joint motion to cause deformation along the sagittal plane of the ankle-foot orthoses (AFOs) [9]. The resulting forces were measured and used to calculate the moment of resistance of the AFOs on the device without taking the joint velocity into account. Luo and coworkers adapted a machine developed for* in vivo* measurement of foot movement to investigate the stability of golf footwear in the frontal plane [10]. The shoes were mounted tightly onto a prosthetic foot during measurement, and identical inversion and eversion torques were applied. They showed an influence of boot shaft stiffness on biomechanical gait parameters and its importance for push-off. In order to not only access two-dimensional movements but also investigate all degrees of freedom at the ankle joint, Böhm and Hösl [11] used an instrumented cable to apply force onto a prosthesis inserted into the boot. Positional data was recorded using a motion capture system while the direction of the applied force was rotated around the boot, which allowed the stiffness of the shaft to be measured without axial loading due to body weight. Similarly, Cappa and coworkers [12] used a robot to move the base of an AFO. The top of the orthosis was connected to a load cell by a universal joint. By moving the base along defined radial lines, the stiffness properties of the plastic orthosis in these directions could be assessed. This setup was the second version of the device, after the first one was used to measure the stiffness in the sagittal and frontal planes only [13]. Although the support provided to the ankle is clearly critical during the loaded phases of gait, to our knowledge, no device has yet been able to measure the RoM of the ankle joint that occurs due to the maximal moments and forces that act during the stance phase of walking.

In order to match the axes of the ankle joint, the RoM clearly needs to be measured in both the sagittal (plantar flexion/dorsal flexion) and frontal (inversion/eversion) planes, while rotations are applied around the ankle joint axes. For ideal testing conditions, the loading rate and peak torque application should be kept constant, in order to ensure the boots are not overstrained during the trial. The fixation of the boot to the last should also be firm enough to prevent movement between the boot and the last. Furthermore, in order to ensure standard testing conditions, tension in the lacing should be objectively verifiable. As the RoM of interest occurs mainly during stance phase, the foot needs to be loaded with body weight (BW) during the measurement. Finally, all measurement procedures should occur in an appropriately rapid manner so as to replicate the dynamic nature of gait and minimize the possible influence of any viscoelastic material behaviour to ensure a fast prototype test phase.

Based on these requirements, the primary aim of this study was to assess the capability of a novel custom device to produce and apply a specific torque to stability boots and to assess ankle joint angle measurements in anatomically suitable axes. The second aim was to determine the reliability of the fast testing method (FTM).

## 2. Methods

### 2.1. Device

**Figure 1:**
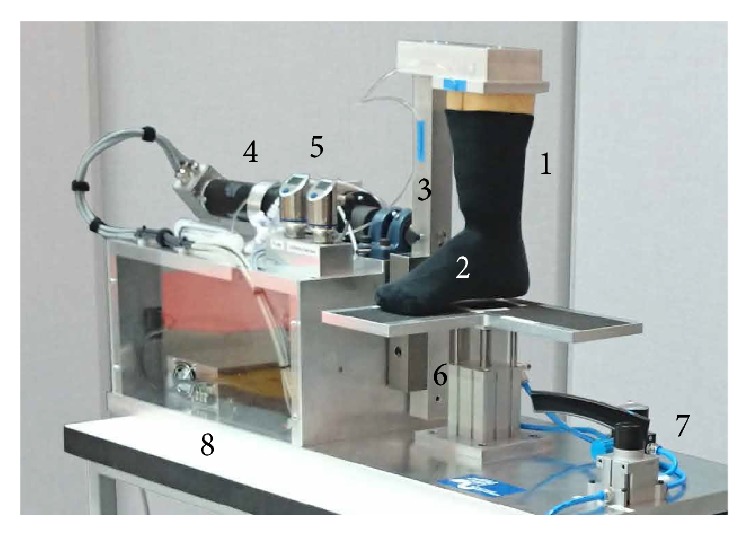
Setup of the fast testing method (FTM) in the flexion/extension position including (1) the wooden shank, with the shank air pad, (2) the last with the instep air pads, (3) the moving arm to apply the torque, (4) the motor, (5) the pressure sensors, (6) the pneumatic cylinder to apply the axial load with the valve (7), and (8) the electronics. The lower limb (1 and 2) is rotated by 90 degrees for inversion and eversion testing.

The requirements for the FTM were the application of axial loading in the range of body weight, together with ankle joint moments that are representative of gait, while simultaneously measuring the ankle joint angle in the sagittal and frontal planes. A FTM device, measuring 400 × 1000 × 570 mm, was developed for table mounted operation, controlled by a custom user interface on a laptop (Figure 1). In order to operate properly, an electrical power source as well as pressurized air was necessary. The structural components of the FTM device were constructed of solid sheets of aluminium in order to reach sufficient stability whilst allowing easy mechanical handling. The main structure consisted of a base plate, two sides, and a lid. The combination of an electric motor (EC60 Number 167132, Maxon Motors AG, Sachseln, Switzerland), a planetary transmission (GP 81 A Number 110413, Maxon Motors AG, Sachseln, Switzerland), and a sensor/encoder (Encoder HEDL 9140 Number 137959, Maxon Motors AG, Sachseln, Switzerland) was screwed onto the structure and used as a drivetrain. A moving arm was used to rotate the wooden shank and foot and thereby create an axial torque around the ankle. The sensor was used to record the angle of deflection of the moving arm. A rigid flange coupling (GMDS038F19F20, SIT (Schweiz) AG, Sirnach, Switzerland) connected the drivetrain with the moving arm by an axle that was kept aligned by two ball bearings (SY504M, SKF, Gothenburg, Sweden).

The shank was custom-made according to an anthropometrical model and the ankle joint was placed using data of Wunderlich and Cavanagh [14], while a wooden last (mechanical form that has a shape similar to that of a human foot) of size 43 was used as the foot for boot insertion during testing. A joint attached to the last, representing the metatarsophalangeal joint, allowed physiologically similar movement in the forefoot region as well as facilitating mounting of the boots. To further enable mounting of the boot, the front part of the toes was removed. The foot and shank were connected by a ball and socket joint to represent the ankle. The position of the joint centre was chosen according to the work of Bruening and coworkers [15] and Isman and Inman [16]. To allow sufficient RoM in the ankle joint, the wood of the last was trimmed around the ankle and the open space filled with foam rubber. On the opposite side of the moving arm, two additional steel bars were attached to act as a counterbalance to the shank and foot components and align the centre of mass of the whole moving arm with its rotation axis.

To generate the axial force representing body weight, a pneumatic actuator was placed below the foot (Kompaktzylinder ADNGF-63-50-P-A, Festo, Esslingen am Neckar, Germany) to push up a platform, which was made of aluminium and coated with sandpaper to increase friction. The pressure was set to 2.50 ± 0.01 bar in order to reach an applied force of 800 N, representing the typical vertical ground reaction force of a person during normal gait [17]. To ensure a consistent position of the boot on the platform, the outline of the sole was marked and the boot aligned before testing started.

A USB cable was used to connect the FTM device to the laptop (ProBook 4545s, Hewlett-Packard, Palo Alto, USA) that controlled the FTM device using a custom user interface (LabVIEW 12.0.1f4, National Instruments, Austin, USA).

### 2.2. Motor: Homing and Relationship between Torque and Current

The rotation of the moving arm was controlled by the maximal current fed to the motor, which was proportional to the torque output produced by the motor at the axle. To determine the torque output at a given current, a spring scale (PESOLA AG, type 80005, precision ±0.03%, read out in 0.25 N steps) with one end fixed was attached to the moving arm. The minimal measureable moment using this spring was 0.057 Nm. After starting in a relaxed position, the motor rotated the moving lever arm upwards, stretching the spring scale and creating a restricting torque, but with a predefined maximum current. At a certain point, the restricting torque reaches the same magnitude as the maximal torque produced by the motor, leading to a balanced stagnation loading. At this point, the sum of the forces on the spring scale, including the weight force of the spring scale and the sling multiplied by the lever arm, resulted in the torque output of the motor at this specific current. By measuring several trials with different currents, the coherence between the current fed into the motor and the torque output (the sensitivity) could be determined. This allowed the required torque to be applied for each physiological movement.

To define the required torques at the ankle for each of the four physiological movements, flexion, extension, inversion, and eversion, gait analysis with an experimental setup adapted from [18–20] of two subjects wearing one of the stability boots was performed. The resulting ankle joint angles exhibited a similar magnitude to those measured in previous studies [21] and were used as a reference for testing the stability boots using the FTM. In several trials, the maximum current fed to the motor was altered until the moving arm stagnated at the required deviation angle. The specific current was determined for each anatomical movement (Table 1) and for the measurements.

The homing procedure was performed as follows: after switching on the FTM device, a metal pin was inserted into a hole drilled into the sidewall close to the moving arm. The moving arm was then rotated until it hit the pin. The resistance of the pin triggered the moving arm to rotate an exact angle (33.3°) in the opposite direction, leading to a precise vertical position.

### 2.3. Consistency in Shoe Pressure due to Lacing

In order to ensure consistency of the boot fixation to the device and tension of the lacing, pressure sensitive air pads were placed on the instep of the foot and the dorsal surface of the shank and fastened with double sided adhesive tape. The two air pads were custom-made (Pearltec AG, Schlieren, Switzerland) for this application. The pads were connected to precision pressure sensors (DELOS SI (405052), JUMO GmbH & Co., Fulda, Germany). A four-way stopcock was connected in series to allow inflation and deflation of the pads using a medical syringe. A sock was pulled over the last and shank components covering the air pads and securing their position, to facilitate the mounting of the boots.

In addition to the normal measurement protocol described in the following chapter, two control protocols were performed three times each in order to test the consistency of the measured pressure in the air pads. The first one (*c*_1_, no movement) left out the movement, while the rest of the procedure remained unchanged. The second control protocol (*c*_2_, sock only) solely inflated the air pads as in the measurement protocol. However, no shoe was mounted and no movement was performed. Both control protocols lasted 30 min, in order to be consistent with the measurement protocol.

### 2.4. Testing Procedure

In total, six different stability shoes were tested. For each shoe the following procedure was performed: The air pads were actively drained of all the air using the syringes (pressure = 0.00 bar). Then they were filled until a pressure of 0.16 bar ± 0.01/0.13 bar ± 0.01 was reached in the neutral position with only the sock on. After mounting the boot, the platform with the pneumatic actuator was raised while making sure that the sole of the shoe was properly fitted inside. Then the laces were tightened such that a pressure of 0.32 bar ± 0.02/0.25 bar ± 0.02 was reached in the air pads.

The first movement tested was plantar flexion. Prior to every new direction, the FTM device bent the shoe over a larger RoM than during normal testing, five times. This ensured that the wooden foot and shank components, as well as the pressure balloons, were optimally distributed within the boot. It also helped to break in the shoes, as they were brand new, in order to reduce hysteresis behaviour. Pilot measurements without this break-in showed a continuously increasing RoM within the first few trials. After these preparation trials, the current feed was set to achieve the appropriate torques and the deflection angle was recorded at stagnation for each measurement trial. This complete procedure was repeated five times resulting in five measurement trials for each shoe.

The whole procedure, including break-in, was then repeated in the opposite direction to simulate dorsal extension. After these two movements in the sagittal plane, the platform was lowered. The shank component was unscrewed from the movement arm and rotated 90° before reattaching it and elevating the platform again. The procedure was then repeated for the testing movements in the frontal plane (eversion/inversion).

After all trials with one boot, the remaining pressure in the air pads was noted, before the platform was lowered. The movement arm was then rotated 90° again after unlacing the boot so it could be removed. Once in the vertical position, the pressure in the air pads was noted one more time. These two pressure measurements were used to ensure consistent behaviour in all the boots. In the end, the air pads were deflated again, to avoid stretching. The entire testing procedure lasted less than 30 minutes for one boot.

### 2.5. Reliability

To assess the reliability of the FTM, the intraclass correlation coefficient ICC(3, *k*), for two-way mixed average measures, was determined over all 24 measurements using the SPSS software suite (IBM, Armonk, USA). This ICC was chosen because each measurement was performed by the same rater and was calculated by using the average of the five measurement trials. The change in the mean from the highest to the lowest value within one measurement series consists of both a random and a systematic contribution and was identified according to Hopkins [22]. In addition, the typical error was calculated as a coefficient of variation [22].

## 3. Results

### 3.1. Relationship between Torque and Current

**Figure 2:**
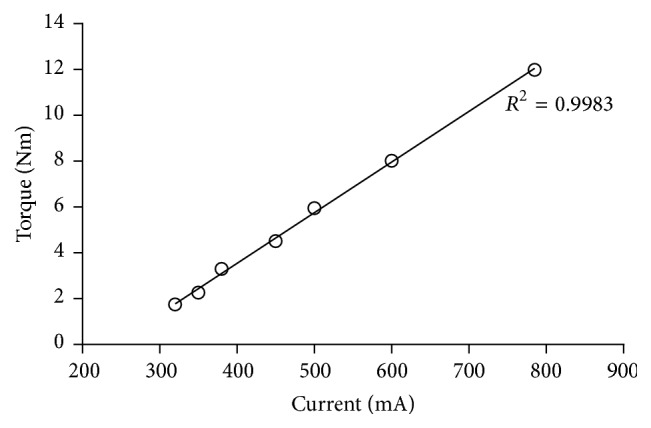
Relation of the current [mA] feed to the motor and the resulting output torque [Nm] including the linear regression (*y* = 0.0221 × −5.2961).

Seven different torques (0.86–11.10 Nm) were used to determine the current input to the torque output of the motor (Figure 2). The outcome showed a linear coherence (*R* ^2^ = 0.9983) between the two measures and enabled the torque output to be controlled by the current fed to the motor. The observed sensitivity was 22.1 Nm/A. On this basis the maximal current input for each anatomical movement was defined (Table 1) using a standard ortho shoe (Künzli AG, Switzerland).

### 3.2. Consistency of Lacing Pressure

**Figure 3:**
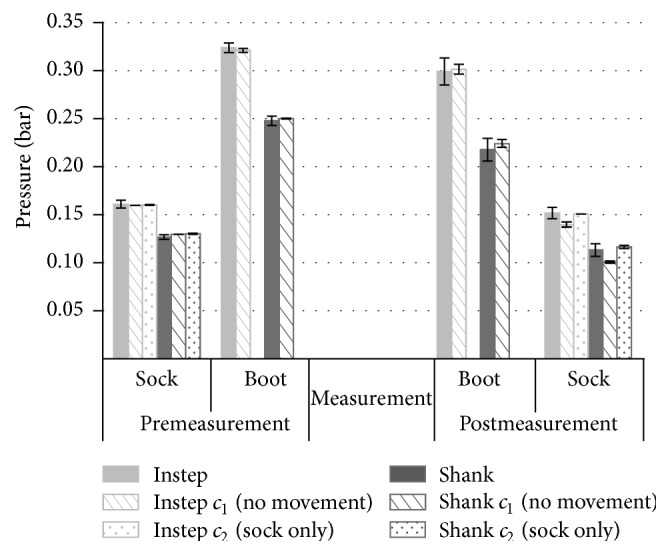
Pressure prior to mounting the boot (premeasurement, sock), after lacing the boot (premeasurement, boot), after the measurement but still with the laced boots (postmeasurement, boot), and after removal of the boot (postmeasurement, sock) for the three different measurement protocols (normal; *c*_1_ (no movement); *c*_2_ (sock only)) measured using both air pads.

For the six measurements, the average pressure with only the sock on (premeasurement, sock) was 0.72% higher than the requested pressure (0.160 bar) on the instep and 2.36% lower than the requested pressure (0.130 bar) on the shank (Figure 3). After lacing (premeasurement, boot), the average pressure was 1.23% higher than the requested pressure (0.320 bar) on the instep and 0.81% lower than the requested pressure (0.250 bar) on the shank (Figure 3). Furthermore, the measurements showed that for all conditions and protocols the pressure decreased over the time period (less than 30 min) of the measurement. The mean decrease in pressure during the six measurement sessions was 7.6/5.5% on the instep (boot/sock) and 12.1%/10.7% on the shank (boot/sock). The pressure decrease without the movement but with the boot (measurement protocol *c*_1_) was −12.5% (instep) and 22.3% (shank). The pressures in the measurement protocol *c*_2_ (sock only) decreased by 5.8% (instep) and 10.5% (shank) on average. During all three protocols, the pressure values showed a similar behaviour over the entire measurement (Figure 3).

### 3.3. Reliability

The measured ankle angles ranged from 3.4° to 25° while the change of the mean was 1.5°. The measured angles resulted in an ICC of 0.99 (95% confidence interval bounds). The standard deviations of the measurement sessions did not change systematically with the size of the measurements and were always <9.8% (Table 2).

Comparing the single trials to each other resulted in a typical change in the average of the ankle angle of 0.01 and a mean typical error of 0.02 (Table 3).

## 4. Discussion

The aim of this study was to assess the capability of a novel custom-built device to apply a reliable and appropriate torque to stability boots, in order to ensure that functional testing can be performed under standardised conditions. One critical aspect of testing such boots is that the lacing pressure must be consistent across all measurements, a problem that has, to date, not yet been sufficiently solved.

### 4.1. Motor: Relationship between Torque and Current

The linear correlation of the input current and the measured moment allowed the output moment to be interpolated and therefore accurately controlled based on the input current to the motor, limited to the range covered by the assessment. Although the procedure used was not a calibration against absolute gauged weights, the relative process to assess the relationship between the torque and the applied current did allow the application of the required torques throughout the measurements. It is important to note that the elements between the motor and the movement arm such as the transmission, rigid flange, coupling, and bearings may have affected the actual moment output on the movement arm. However, demonstrated high correlation (*R* ^2^ = 0.9983; Figure 2) provided confidence that the effects of these intermediate elements were consistent and therefore had little or no impact on the resulting output torque. Furthermore, the motor showed consistent output values in both directions. As a result, the direction of the movement could be easily controlled by the sign of the current, while the value of the torque was defined by the current input (Table 1).

### 4.2. Consistency of Lacing Pressure

The novel use of air pressure in the testing setup allowed not only uniform pressure conditions to be achieved around the mounted boot but also shape variations between the wooden last and the boot to be compensated. In addition, this approach enabled the pressure on the foot due to lacing to be controlled and verified (Figure 3). In doing so, we have been able to ensure consistent lacing pressures prior to and during the measurements, thus reducing disturbances due to varying lacing pressures. The lacing pressures were chosen to be in the range where small changes of the air pressure did not significantly affect the RoM. Mounting the boot and measurement movements seems to have influenced the lacing pressure. The pressure in the air pads decreased over the time of the measurements, possibly due to leaking, loosening of the lacing, or adjustment of the position of the air pads within the boot. However, due to the short testing time of the approach presented, no influence on the measurement results was observed.

### 4.3. Reliability

Our findings indicate that the fast testing method can quantify the ankle joint's RoM in stability boots with good reliability. Therefore, it is an appropriate method to measure differences between various models of stability boots. Furthermore, this testing approach can be applied to evaluate modifications and prototypes during renewal and innovation processes.

The observed errors were most likely caused by two different aspects: a systematic and a random contribution. The systematic error has several sources, mainly the axial load (pressure sensor), the sensitivity of the motor, and the measurement of the ankle angle. The random error is likely due to the position of the shoe and the behaviour of the shoe during the trials, and its effect could certainly be reduced by testing the shoe several times.

While the FTM is not able to replace subject testing completely, it can reduce patient time for testing and improves efficiency during the development of new boots. Furthermore, this method allows for objective comparisons between different boots, which has not been possible to this extent previously, with subject testing only.

### 4.4. Outlook

In the healthy foot, a higher stiffness of the boot does not equate with safety [11]. These authors suggest a trade-off between lateral stiffness and free natural motion of the ankle joint complex. A modern shoe production allows a change of the stiffness dependent upon the direction of the motion. In the future, the presented approach could allow manufacturing subject specific shoes based on individual requirements, for example, during rehabilitation, after injury, or for protection. Furthermore the mechanical aspects could be improved with proprioception elements, similar to the manner in which the active ankle stiffness can be influenced by proprioception [23].

## 5. Conclusion

The FTM device has been able to measure the ankle joint RoM in both the sagittal (plantar flexion/dorsal flexion) and frontal planes (inversion/eversion). Determination of the relationship between output torque and the current applied to the device was successful and allowed for an appropriate torque to be applied throughout the measurements. Fixation of the boot on the last was consistent for all the measurements and tight enough to prevent last-to-boot movement. Body weight was taken in account by the pneumatic actuator. All the measurements were completed in less than 30 minutes. The measurement results were shown to be reliable. Such an approach now allows the fast and reliable testing of subject specific manufactured shoes with a motion dependent stiffness of the shaft.

## Figures and Tables

**Table 1:** Appropriate input current [mA] and resulting torque [Nm] for the requested joint angles.

Movement	Joint angle [°]	Input current [mA]	Torque [Nm]
Plantar flexion	3.5	−380	−3.1
Dorsal extension	14.5	785	12.1
Eversion	3.5	−320	−1.8
Inversion	7.0	450	4.6

**Table 2:** Mean ankle angles and standard deviations (SD) from all 24 measurements sorted by anatomical movements (^*∗*^device reached maximal deflection of the test device due to low stiffness of the shoe).

Shoe	Flexion		Extension		Eversion		Inversion	
	[°]	SD	[°]	SD	[°]	SD	[°]	SD
1	3.68	0.19	14.60	0.20	3.74	0.09	6.58	0.31
2	5.06	0.17	10.72	0.29	3.40	<0.01	7.24	0.15
3	3.70	0.12	22.96	0.11	3.88	0.08	8.72	0.24
4	4.32	0.26	17.48	0.04	3.64	0.09	8.68	0.08
5	4.20	0.20	24.58	0.18	6.26	0.61	25.00^*∗*^	<0.01^*∗*^
6	3.66	0.09	14.56	0.15	3.78	0.08	6.46	0.36

**Table 3:** Comparing the single measurement trials to each other.

Trial comparison	Change in the average [°]	Typical error [°]
2-1	−0.01	0.03
3-2	−0.01	0.02
4-3	−0.01	0.01
5-4	−0.01	0.01
